# Access to maternal health services for young women with disabilities in Sub-Saharan Africa: a scoping review protocol

**DOI:** 10.1136/bmjopen-2025-106638

**Published:** 2025-10-20

**Authors:** Pebalo Francis Pebolo, Emmanuel Kimera, Faustine Kyungu Nkulu Kalengayi, Fredinah Namatovu

**Affiliations:** 1 Department of Epidemiology and Global Health, Umeå University, Umeå, Sweden; 2 Reproductive Health, Gulu University, Gulu, Gulu, Uganda; 3 Mountain of the Moon University, Fort Portal, Uganda

**Keywords:** Maternal medicine, Disabled Persons, Health Services Accessibility, Delivery of Health Care, Integrated

## Abstract

**Abstract:**

**Introduction:**

Young women with disabilities (WWDs) face multiple barriers in accessing maternal healthcare services in low-resource settings. Consequently, they are at an increased risk of adverse maternal health outcomes due to young age and having a disability. This review focuses on synthesising evidence regarding the extent of access to maternal healthcare services and the barriers faced by young WWDs in Sub-Saharan Africa.

**Methods and analysis:**

We will conduct a scoping review guided by the updated Joanna Briggs Institute methodology for scoping reviews. A systematic search of MEDLINE, EMBASE, Scopus, CINAHL, Web of Science Core Collection, Global Health, African Journal Online and Women’s Studies International will be performed to identify relevant articles published in English from 2007 to 2025. A team of two reviewers will independently screen the retrieved articles for relevancy based on the inclusion criteria, and a thematic synthesis will be undertaken to develop a descriptive analysis.

**Ethics and dissemination:**

Since this review will only involve the analysis of published data, it does not require ethical approval. The results will be published in a peer-reviewed journal.

**Registration:**

This review has been registered with the Open Science Framework DOI; https://doi.org/10.17605/OSF.IO/Q7Y8S.

**STRENGTHS AND LIMITATIONS OF THIS STUDY**

- The protocol includes a robust search across eight scholarly databases using Population, Concept and Context (PCC) framework to ensure wide coverage of relevant literature, capturing diverse and context-specific studies, notably from Sub-Saharan Africa.
- The structured approach for identifying, selecting, charting and synthesising evidence, using the updated Joanna Briggs Institute guidelines, enhances the reliability and reproducibility of our findings.
- As with any study, despite the data being sourced from peer-reviewed published papers, there remains a risk of bias that could be transferred to this review from primary sources.
- Limiting this review to peer-reviewed articles published only in English between 2007 and 2025 means that evidence about access to maternal healthcare services for young women with disabilities published in other languages, outside the specified period and non-peer-reviewed sources will be excluded.

## Introduction

 Globally, there has been significant improvement in maternal health in the last two decades.^1^ This improvement resulted from initiatives such as the United Nations’ Sustainable Development Goals (SDGs)^2 3^ and the Universal Health Coverage, a human rights approach to equitable access to sexual and reproductive health services.^2 4^ The SDG strategies on maternal health offer an opportunity for the international community to work together and accelerate progress to improve the health of all women, in all countries, under all circumstances.^5^ However, despite this global commitment, vulnerable populations such as women with disabilities (WWDs) have limited access to these essential maternal health services.^6^

In this study, disability is defined as a structural or functional impairment that limits an individual’s full participation in societal activities on an equal basis with others.^7^ WWDs face multiple deprivations and are vulnerable in experiencing healthcare services since the access is constructed through ableism—a societal system that favours able-bodied individuals while discriminating against those with disabilities.^8^

WWDs encounter more obstacles in accessing maternal healthcare as they do with general healthcare. These challenges include negative attitudes from health workers, limited transportation options for travelling to healthcare facilities and a lack of accessible health information.^4 9^ Tackling these inequalities is seen as an essential opportunity to guarantee equitable and respectful maternity care to all^2^ and minimise poor maternal outcomes among WWDs including young women with disabilities (YWWDs).

Despite the significant research output recently on maternal health for WWDs in the Western World,^10^ low- and middle-income countries are underrepresented, yet more than 80% of the estimated 1.6 billion persons with disability live in these settings.^4^ The pattern of discrimination among WWDs varies based on individual factors such as the type and number of disabilities, age,^11^ the socio-economic status and variation in family support systems.^12^ Additionally, WWDs encounter health systems barriers such as providers’ attitudes and physical accessibility.^13^ The intersection of these individual and health systems factors among YWWDs^14 15^ worsens access to vital maternal healthcare services. This is particularly worse where healthcare is primarily financed through out-of-pocket expenditure,^16 17^ driving a number of YWWDs to resort to home delivery, which is prone to complications.^18^ In some cultures, pregnant young girls with disabilities are forced to abort, marry or live with the baby’s father to raise the child together.^19^

As stated earlier, the intersectional barriers between the individual and health system factors^11 17 20^ pose difficulty in accessing maternal health services to YWWDs^17 21^ in low-income countries. In Uganda, implementation of disability inclusive legislation and policy is faced with numerous technical and financial challenges; hence, WWDs experience multiple physical, attitudinal, communication and structural barriers to access and use of sexual and reproductive health services.^22^ Additionally, the approach to maternal healthcare for WWDs differs based on the types and number of disabilities.^23 24^ For example, women with hearing disabilities report longer hospital stays compared with those with other forms of disabilities.^25^ Women having physical disabilities with altered anatomical makeup encounter difficulties in standard obstetrics or gynaecological assessments such as pelvic examinations.^26^ Vital communications such as consultation, counselling, health education and prescription instructions that are often experienced during clinical encounters are not often accessible to those with hearing impairments,^26^ which compromises the clinical decision-making process during maternal healthcare.

Institutional challenges such as inappropriate infrastructures and distance to the nearest maternal healthcare service facilities pose physical inaccessibility to YWWDs.^27^ Moreover, healthcare providers tend to treat YWWDs in a stereotyped fashion, which increases the risk of prescribing caesarean sections and induction of labour in the absence of standard obstetrical indications.^28^ Additionally, maternal healthcare services such as labour and delivery for YWWDs are more demanding and often require the presence of a senior healthcare provider such as a medical doctor.^29^ An understanding of the experiences of YWWDs in accessing maternal healthcare is important to ensure the needs of this population are met, a crucial step in informing appropriate strategies to improve disability inclusive maternal healthcare services in low-income settings. A preliminary search of MEDLINE, the Cochrane Database of Systematic Reviews and Joanna Briggs Institute *(JBI) Evidence Synthesis* was conducted and no current or underway systematic or scoping reviews on extent and barriers to access of maternal healthcare services for YWWDs in Sub-Saharan Africa (SSA).

### Aim, objectives and research questions

The overarching aim of this scoping review is to synthesise evidence on access to maternal healthcare services for YWWDs in SSA. By synthesising evidence on the current state of knowledge on access to maternal healthcare services, we will identify needs and priorities required to inform policies and programmes and future research in SSA. This review will strive to answer the question related to the extent of access to maternal healthcare services and barriers faced by YWWDs in SSA.

## Methods and analysis

This review will follow the updated Joanna Briggs Institute (JBI) methodology for scoping reviews.^30^ The JBI guideline was developed based on the methodological framework for scoping reviews proposed by Arksey and O’Malley.^31^ This involves identifying the research question and relevant studies, selecting the studies, charting the data and reporting the findings. We consider this as the most appropriate way to synthesise evidence on access to maternal healthcare services for YWWDs in SSA. The updated JBI guidelines not only propose a detailed and comprehensive step-by-step process of conducting scoping reviews, but they are also highly recommended to ensure a high-quality review in a clear and transparent manner.^30^

This scoping review will be reported according to the Preferred Reporting Items for Systematic Reviews and Meta-analyses extension for Scoping Review checklist (PRISMA-SR).^32^ The following steps will be used to conduct the reviews: (1) search strategy, (2) study selection, (3) charting the data, (4) collating, (5) summarising and (6) reporting the results. This review has been registered with the Open Science Framework DOI; https://doi.org/10.17605/OSF.IO/Q7Y8S.

### Search strategy/structure

The search strategy will be developed using the Population, Concept and Context framework to systematically break down the searchable elements of the research question, a method commonly employed in scoping review searches. Keywords and controlled vocabulary search terms (online supplemental appendix 1) will be used with the boolean operator (AND/OR) alongside Medical Subject Heading terms to refine the search string. The syntax created will be adjusted based on each database’s requirements. Geographic search terms will be used to focus search retrieval on articles referencing SSA countries. The results of the search and the study inclusion process will be reported in full in the final scoping review and presented in a PRISMA-SR flow diagram.^32^ Example search string: (*“young women” OR “female youth” OR “adolescent girls”) AND (“disabilities” OR “disabled persons” OR “physical disability” OR “hearing impairment”) AND (“maternal health services” OR “antenatal care” OR “postnatal care” OR “pregnancy care” OR “labour and delivery”) AND (“Sub-Saharan Africa” OR “Africa South of the Sahara”*).

### Information sources

Literature search will be conducted using eight scholarly databases: MEDLINE (via Ovid interface), EMBASE (via Embase.com), Scopus, CINAHL (via EBSCOhost), Web of Science Core Collection (via Thomson Reuters), Global Health (via CABI), PsycINFO (via EBSCOhost), African Journal Online (AJOL) and Women’s Studies International (via EBSCOhost). We believe that these eight scholarly databases are most widely used and are comprehensive in health literatures.

### Study selection

We will include studies conducted among women with any form of disabilities between ages 15 and 24 years. The definition of maternal health dimensions will include pregnancy, antenatal care, labour and delivery, and postnatal care as indicated in the keywords (online supplemental appendix 1). The included studies should report the views, perceptions and perspectives of YWWDs on access to maternal healthcare services. Studies involving mixed age groups, including those below 15 and/or above 24 years will be included if data for the age groups 15–24 years has been reported separately. We will exclude studies exclusively focusing on those older than 24 years and those younger than 15 years of age. This ensures a clearer understanding of YWWD’s specific maternal health needs. We will include studies with data from multiple countries if SSA country-specific data were reported separately. Qualitative, quantitative and mixed-method peer-reviewed studies published in English will be included. This scoping review will cover studies conducted in SSA from 1 January 2007 to 30 June 2025. The 2007 start date was chosen to identify changes in access to maternal healthcare services from the initial Maputo Plan for action 2007–2015 and the revised Maputo Plan of action 2016–2030 (the post 2015 plan of action).^33^ Technical reports, theses, research protocols, book reviews, quasi-experiments, randomised control trials, conference proceedings, commentaries, blog posts and other kinds of grey publications will be excluded as this will keep the relevance of the reviews.

### Screening, charting and collating data

The titles and abstracts of the identified articles will be screened to eliminate those that are obviously irrelevant to the review. This screening will be done by two independent reviewers using the Rayyan web-based tool.^34^ Thereafter, full texts for selected references will be printed to assess their eligibility based on the inclusion and exclusion criteria. Eligibility assessment will be done by two reviewers working independently, and any disagreement will be resolved by consensus involving the third reviewer. Reasons for the exclusion of sources of evidence in full text that do not meet the inclusion criteria will be recorded and reported in the scoping review.

Data will be extracted from the selected articles using a pre-designed and pre-tested Microsoft Excel data extraction form. This will include the study citation, countries and settings, population, aims, sample size, key findings related to the extent of maternal health healthcare access, and barriers faced by YWWDs. The draft extraction form provided (online supplemental file appendix 2) will be modified and revised as necessary during the process of data extraction and the modifications will be detailed in the scoping review report.

### Data summary, analysis and presentation

Descriptive summary of the data will be presented in terms of figures, tables and themes. The data will be deconstructed and reconstructed in a thematic analysis. This will enable us to present a narrative account of all extracted information from the articles based on overlaps and diversities of the findings to answer our research questions.

## Ethics and dissemination

Since this review will only involve the analysis of published data, it does not require ethical approval. On completion of the scoping reviews, we anticipate that these results will be disseminated through publication in a peer-reviewed journal and conferences targeting researchers, programmers and policymakers. The data set will be made available at Umea University, Gulu University, and Mountain of the Moon University. Findings from this scoping review are expected to open a discussion on access to maternal health services and provide a foundation/agenda for future research on maternal healthcare for young women with disabilities in Sub-Saharan Africa.

## Supplementary material

10.1136/bmjopen-2025-106638 online supplemental file 1

10.1136/bmjopen-2025-106638 online supplemental file 2

## Data Availability

No data are available.
