# Supplementary material for: Access to maternal health services for young women with disabilities in Sub-Saharan Africa: a scoping review protocol
Source: BMJ Open. 2025 Oct 20;15(10):e106638. doi: 10.1136/bmjopen-2025-106638 (PMC12542724; doi:10.1136/bmjopen-2025-106638)
Supplement: online supplemental file 1 [file bmjopen-15-10-s001.docx]

**Appendix I: Search Strategy and Research Question**

**Keywords used**

- Maternal Health
- Maternal Healthcare
- Maternal Health services
- Pregnancy Care
- Antenatal Care
- Postnatal Care
- Childbirth care
- Labor and delivery care
- Disabled Person(s)
- Women with Disabilities
- Sub-Saharan Africa
- Africa South of the Sahara
- Health Services Accessibility
- Nursing Care
- Obstetric Care
- Cesarean section
- Normal Delivery
- Health Education
- Unplanned Pregnancy
- Postpartum Family Planning Services

**Research question**

What insights does the literature provide on experiences, perceptions, and perspectives related to access to maternal healthcare services for female youth with disability in Sub-Saharan Africa?
