# Supplementary material for: Access to maternal health services for young women with disabilities in Sub-Saharan Africa: a scoping review protocol
Source: BMJ Open. 2025 Oct 20;15(10):e106638. doi: 10.1136/bmjopen-2025-106638 (PMC12542724; doi:10.1136/bmjopen-2025-106638)
Supplement: online supplemental file 2 [file bmjopen-15-10-s002.docx]

**Appendix II: Data Extraction Template**

| **S/No.** | **First authors surname (year)** | **Country** | **Study type** | **Study Population** | | **Study Objective(s)** | **Maternal Health care Access** | | | **Key findings** |
| --- | --- | --- | --- | --- | --- | --- | --- | --- | --- | --- |
|  |  |  |  | **YWWD** | **Maternal Health Providers** |  | **Pregnancy and Antenatal care** | **Labor and Delivery** | **Postnatal care** |  |
|  |  |  |  |  |  |  |  |  |  |  |
