# Supplementary material for: Access to maternal health services for young women with disabilities in Sub-Saharan Africa: a scoping review protocol
Source: BMJ Open. 2025 Oct 20;15(10):e106638. doi: 10.1136/bmjopen-2025-106638 (PMC12542724; doi:10.1136/bmjopen-2025-106638)
Supplement: Supplementary file 3 [file bmjopen-2025-106638.reviewer_comments.pdf]

PEER REVIEW HISTORY

BMJ Open publishes all reviews undertaken for accepted manuscripts. Reviewers are asked to complete a checklist review form and are provided with free text boxes to elaborate on their assessment. These free text comments are reproduced below.

ARTICLE DETAILS

Title (Provisional)

Access to Maternal Health Services for Young Women with Disabilities in Sub-Saharan Africa: A Scoping Review Protocol

Authors

Pebolo, Pebalo Francis; Pebolo, Pebalo Francis; Kimera, Emmanuel; Nkulu Kalengayi, Faustine Kyungu; Namatovu, Fredinah

---

VERSION 1 - REVIEW

**Reviewer**	**1**
**Name**	**Opoku, Daniel**
**Affiliation**	**Technische Universitat Berlin, Health Care Management**
**Date**	**11-Aug-2025**
**COI**	**None**

Authors should consider exploring publications in all languages and utilize the advancements in translation software available to aid in the review.

VERSION 1 - AUTHOR RESPONSE

Reviewer 1:

Comment: Authors should consider exploring publications in all languages and utilize translation software.
Response: We appreciate this suggestion and agree with the reviewer regarding the potential value of including non-English studies especially considering the availability of translation software. However, we are concerned about the verification of the accuracy of the output in the absence a native speaker to double check. We worry that automated translations can distort meaning, especially in qualitative research, where subtle language cues are critical. Moreover, academic texts often contain complex terminology, cultural references, and discipline-specific language which may be oversimplified leading to inaccurate or misleading translations. Based on these concerns, we consider English articles as the most suitable for inclusion.
